# Interleukin-10 Overexpression Promotes Fas-Ligand-Dependent Chronic Macrophage-Mediated Demyelinating Polyneuropathy

**DOI:** 10.1371/journal.pone.0007121

**Published:** 2009-09-22

**Authors:** Dru S. Dace, Aslam A. Khan, Jennifer L. Stark, Jennifer Kelly, Anne H. Cross, Rajendra S. Apte

**Affiliations:** 1 Department of Ophthalmology and Visual Sciences, Washington University in St. Louis School of Medicine, St. Louis, Missouri, United States of America; 2 Department of Neurology, Washington University in St. Louis School of Medicine, St. Louis, Missouri, United States of America; 3 Department of Developmental Biology, Washington University in St. Louis School of Medicine, St. Louis, Missouri, United States of America; 4 The National Multiple Sclerosis Society, New York, New York, United States of America; New York University School of Medicine, United States of America

## Abstract

**Background:**

Demyelinating polyneuropathy is a debilitating, poorly understood disease that can exist in acute (Guillain-Barré syndrome) or chronic forms. Interleukin-10 (IL-10), although traditionally considered an anti-inflammatory cytokine, has also been implicated in promoting abnormal angiogenesis in the eye and in the pathobiology of autoimmune diseases such as lupus and encephalomyelitis.

**Principal Findings:**

Overexpression of IL-10 in a transgenic mouse model leads to macrophage-mediated demyelinating polyneuropathy. IL-10 upregulates ICAM-1 within neural tissues, promoting massive macrophage influx, inflammation-induced demyelination, and subsequent loss of neural tissue resulting in muscle weakness and paralysis. The primary insult is to perineural myelin followed by secondary axonal loss. Infiltrating macrophages within the peripheral nerves demonstrate a highly pro-inflammatory signature. Macrophages are central players in the pathophysiology, as *in vivo* depletion of macrophages using clodronate liposomes reverses the phenotype, including progressive nerve loss and paralysis. Macrophage-mediate demyelination is dependent on Fas-ligand (FasL)-mediated Schwann cell death.

**Significance:**

These findings mimic the human disease chronic idiopathic demyelinating polyneuropathy (CIDP) and may also promote further understanding of the pathobiology of related conditions such as acute idiopathic demyelinating polyneuropathy (AIDP) or Guillain-Barré syndrome.

## Introduction

Macrophages carry out a wide variety of biological functions and their ultimate effector phenotype is largely dependent upon activation and polarization. Polarization, in turn, is regulated by the dominant cytokine signature in the resident tissue microenvironment [1], [2]. Macrophages can acquire a “classically-activated” phenotype (i.e. M1 macrophage) and display an anti-angiogenic, anti-bacterial, and pro-inflammatory functions; or an “alternatively-activated” phenotype (i.e. M2 macrophages) and display a pro-angiogenic and anti-inflammatory phenotype. Of the cytokines involved in macrophage polarization, the immunosuppressive cytokine interleukin 10 (IL-10) plays a highly robust role in M2 polarization. IL-10-mediated polarization of macrophages towards an M2 phenotype has a detrimental affect on the ability of macrophages to regulate abnormal angiogenesis as seen in the eye [1], [3], [4]. This has particular relevance to the eye as age-related macular degeneration (AMD), the leading cause of blindness in people over 50 years of age, is characterized by the development of abnormal blood vessels underneath the retina called choroidal neovascularization (CNV). In mouse models of CNV in AMD, IL-10 promotes pathological neovascularization by preventing macrophage infiltration into the choroid and by polarizing macrophages to a pro-angiogenic M2 phenotype [3]. IL-10 has also been shown to promote pathological angiogenesis in the retina following ischemia [4].

Our laboratory was interested in exploiting the pro-angiogenic and anti-inflammatory properties of IL-10 in a model of age-related macular degeneration (AMD). We constructed transgenic mice expressing murine IL-10 under the control of the human VMD2 gene promoter in B6CBAF2/J founder mice [3]. VMD2 is located on chromosome 11q13, and encodes the 585 aa protein Bestrophin. Mutations in VMD2 have been implicated in Best vitelliform macular dystrophy and AMD, and bestrophin was originally identified as being localized to the basolateral plasma membrane of retinal pigment epithelium (RPE) cells [5]. VMD2-IL10 transgenic mice expressed high levels of secreted IL-10 in the retina, and were prone to develop CNV following laser-injury [3]. After creation of VMD2-IL-10 transgenic mice, our laboratory began to backcross these mice to a C57BL/6 background. Surprisingly, IL-10 transgene-positive mice at the F5 backcross generation developed spontaneous hindlimb weakness around 3-months of age, followed by eventual paralysis. Veterinary necropsy of diseased animals revealed swollen peripheral nerves, with necrotic lesions and immune cell infiltrate. This pattern of paralysis continued through subsequent generations of transgenic mice. At the F11 backcross generation, transgenic mice were monitored for development of paralysis twice weekly, using a modified grading scale similar to mouse EAE models [6]. In this study we explore the pathobiology of the autoimmune polyneuropathy associated with transgenic IL-10 overexpression.

## Results

**Figure 1:**
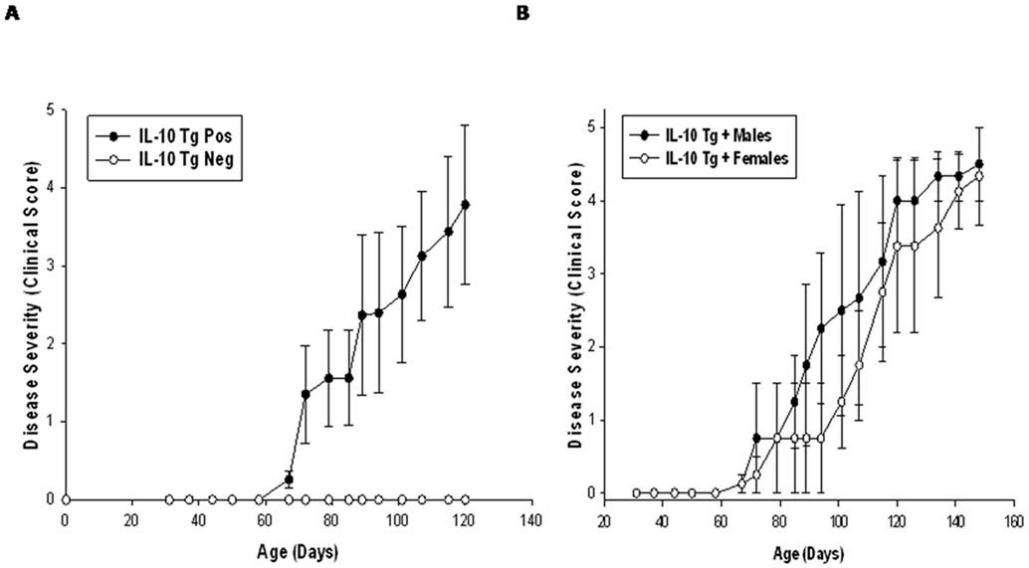
Disease progression in VMD2-IL-10 transgenic mice. A, Tg+ mice (n = 9) develop spontaneous hindlimb paralysis, whereas age-matched Tg− littermates (n = 5) do not develop disease. B, IL-10 overexpression observed in the testes does not significantly exacerbate disease, as male Tg+ mice (n = 5) develop similar progression of disease compared to Tg+ female mice (n = 4).

Mice positive for the IL-10 transgene as determined by PCR analysis developed hind limb paralysis (Fig. 1A). Paralysis was restricted to hind limbs of transgene positive (Tg+) mice, as paralysis of the tail was not observed. Transgene negative (Tg−) littermates did not develop hind limb paralysis (Fig. 1A). Disease onset varied from 2-4 months of age, and Tg+ mice <2 months of age showed no overt loss of motor function.

**Figure 2:**
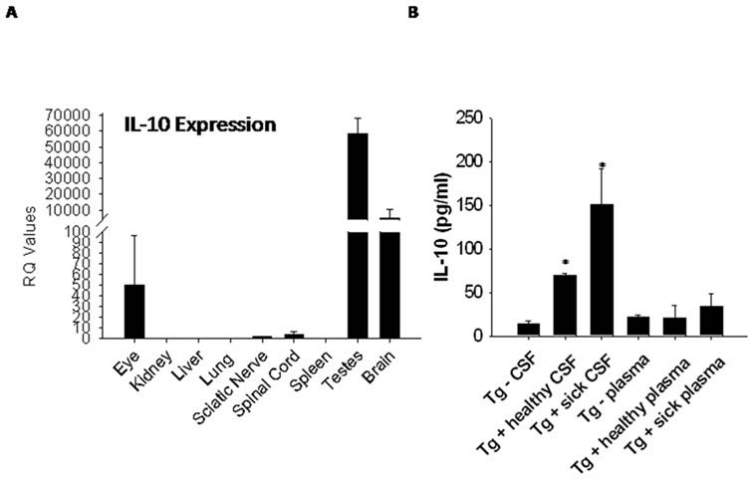
IL-10 gene and protein expression in VMD2-IL-10 transgenic mice. A, Gene expression of IL-10 is upregulated in the eye, testes, brain, sciatic nerve, and spinal cord of Tg+ sick animals compared to tissues from Tg− littermates, as determined by quantitative real-time RT-PCR. B, IL-10 protein levels from the cerebrospinal fluid (CSF) significantly increase in correlation with disease progression (p≤0.002 for each stage of disease), as determined by IL-10 specific ELISA. Plasma levels of IL-10 are not elevated in Tg+ mice.

As stated above, VMD2 was originally identified as being localized to RPE cells. However, subsequent data has shown that human VMD2 is expressed in multiple sites within the human body [7]. GeneAtlas analysis reveals that in humans, VMD2 is highly expressed in the nervous system, including the spinal cord and multiple areas of the brain. For mice, murine VMD2 appears to be primarily expressed in the eye and testes [8]. However, the full expression pattern of human VMD2 within murine tissues is unknown. Since human VMD2 was driving murine IL-10 expression in the Tg+ mice, we examined gene expression of murine IL-10 in multiple tissues from Tg+ sick mice compared to normal Tg− control littermates, as Tg− tissues should express baseline levels of IL-10. We found increased expression of IL-10 in the eye, sciatic nerve, spinal cord, testes, and brain of Tg+ sick mice relative to IL-10 levels in the same tissues from Tg− mice (Fig. 2A). Similar expression patterns of IL-10 were observed in Tg+ healthy mice relative to Tg− mice (data not shown). Based on studies demonstrating high VMD2 expression in the testes, and since IL-10 gene expression was 55,000 fold higher in the testes of Tg+ mice, we sought to determine if increased IL-10 gene expression in the testes of male Tg+ mice may exacerbate development of disease compared to female Tg+ mice. Male Tg+ mice developed a slightly accelerated paralysis compared to female Tg+ mice (Fig. 1B). However, these differences in progression of disease were not significant and ultimately both male and female Tg+ mice developed paralysis.

To determine if IL-10 protein levels were upregulated systemically or localized to the neural tissue micromilieu, plasma and cerebrospinal fluid (CSF) of male and female Tg+ healthy mice, Tg+ sick mice, and Tg− littermates were harvested and examined by IL-10 ELISA. We found a significant increase of IL-10 protein levels in the CSF of Tg+ healthy mice compared to Tg− littermates(p<0.001), with significantly (p = 0.002) increasing levels that correlated with development of disease (Fig. 2B). Plasma levels of IL-10 protein were not elevated in Tg+ mice, indicating that systemic levels of IL-10 do not change and suggesting that localized IL-10 expression was driving development of disease. Additionally, we examined local expression of IL-10 protein by immunohistochemistry in organs directly affected by disease. We observed increased expression of IL-10 on the sciatic nerve of Tg+ healthy and sick mice (Fig. S1B and C). IL-10 staining was not observed on Tg− sciatic nerve sections (Fig. S1A). Spinal cord expression of IL-10 appeared to be localized to the dorsal roots of Tg+ sick mice (Fig. S1F), and was not apparent on Tg− or Tg+ healthy spinal cord tissue sections (Fig. S1D and E).

**Figure 3:**
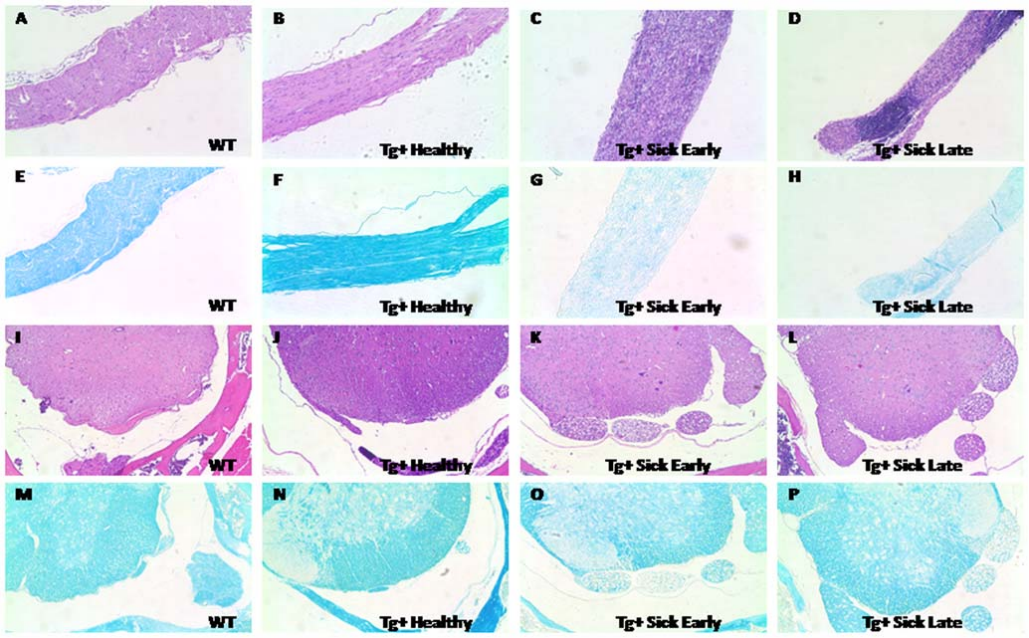
Pathology of disease in VMD2-IL-10 transgenic mice: sciatic nerve sections from mice. H&E staining of the sciatic nerve (A–D) reveals no significant cellular infiltrate in wild-type A, Tg− littermates or B, Tg+ healthy mice. C, Tg+ mice in the early stages of disease (clinical score <3) reveal a significant infiltration of cells into the sciatic nerve. D, Tg+ mice in the late stages of disease (clinical score ≥3) reveal cellular infiltration and loss of tissue diameter. Luxol fast blue staining of the sciatic nerve E–H, reveal no demyelination in (E) wild-type mice or F, Tg+ healthy animals. G,H, Significant demyelination is observed in both Tg+ sick early and Tg+ sick late mice. I–L, H&E staining of the spinal cord reveals no significant infiltration of cells, regardless of stage of disease. M–P, Luxol fast blue staining of the spinal cord reveal no observable demyelination in M, wild-type and N, Tg+ healthy animals. O, Tg+ sick early and P, late mice both demonstrate significant demyelination of the dorsal roots of spinal cord tissue. All images acquired at 100× magnification.

In order to examine the pathology of disease in VMD2-IL-10 Tg mice, histological examination of various tissues from Tg−, Tg+ healthy, and Tg+ sick mice was performed. H&E staining of the sciatic nerve from Tg− and Tg+ healthy mice was normal (Fig. 3A and B), however, Tg+ sick early (defined as having a disease score of <3) sciatic nerve tissue sections revealed a dramatic infiltration of mononuclear cells (Fig. 3C). Tg+ sick late (disease grade ≥3) mice continued to have a dramatic infiltration of mononuclear cells (Fig. 3D), and also exhibited a significant loss of sciatic nerve tissue, as evidenced by a decrease in tissue diameter. Luxol fast blue staining of sciatic nerve tissue revealed no loss of myelin in Tg− and Tg+ healthy mice (Fig. 3E and F). Conversely, Tg+ sick tissues revealed significant loss of myelin staining (Fig. 3G and H). This correlation of cell infiltrate with loss of myelin in the sciatic nerve suggested that disease progression in Tg+ mice was due to an autoimmune attack of the peripheral nerves. The central nervous system, including the brain (data not shown) and the spinal cord itself, seemed resistant to the effects of increased IL-10, as H&E staining of the spinal cord tissue revealed no evidence of cell infiltrate in Tg−, Tg+ healthy, nor Tg+ sick mice (Fig. 3I-L). The dorsal roots of Tg+ sick mice do reveal a slight “moth-eaten” appearance, and some loss of myelin staining was observed in these dorsal roots (Fig. 3O and P). No loss of myelin was observed in the spinal cords of Tg− or Tg+ healthy mice (Fig. 3M and N).

In addition to the sciatic nerve, other peripheral nerves were examined for cellular infiltrate. In nerves from the brachial plexus, significant infiltrate was observed in Tg+ sick mice (Fig. S2C), but not in Tg− or Tg+ healthy mice (Fig. S2A and B). This is interesting as no paralysis was observed in the forelimbs during the course of disease. This suggests that disease in Tg+ mice may be ascending to the forelimbs, as often seen in human CIDP, yet mice perish prior to forelimb paralysis occurs. No cellular infiltrate was observed in the femoral nerve, regardless of disease state (Fig. S2D and F). In the optic nerve, no cellular infiltration was observed in Tg+ sick mice (Fig. S2G), despite the increased expression of VMD2 and IL-10 in the eyes of these animals. A summary of tissues examined, cellular infiltration, and phenotype is displayed in Table S1.

**Figure 4:**
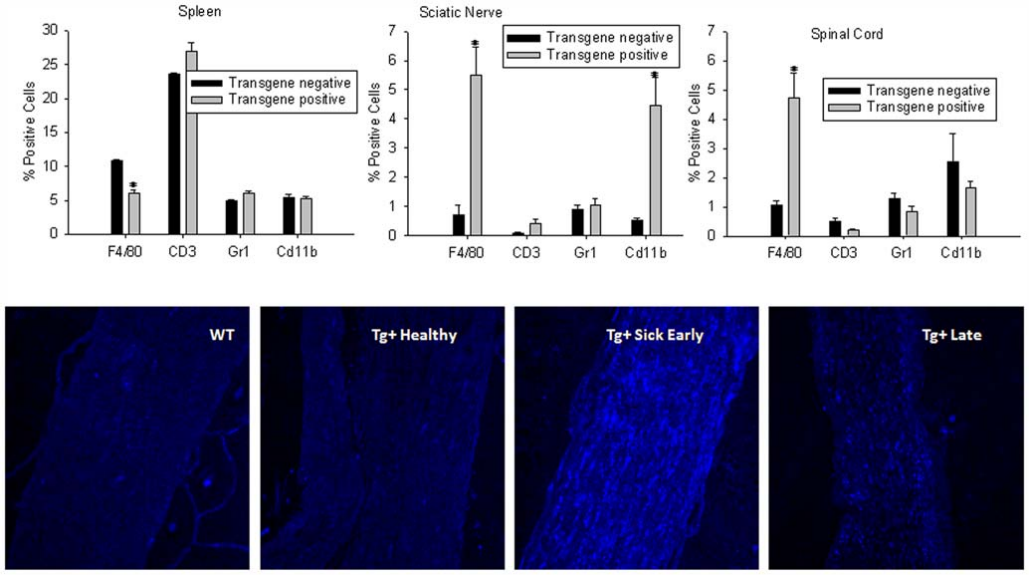
Cellular infiltrate in VMD2-IL-10 transgenic mice. A, Flow cytometry of spleens from animals reveal a slightly decreased presence of F4/80+ macrophages (p<0.001) and increased CD3+ T cells (p = 0.008) in Tg+ sick mice compared to Tg− littermates. B, Flow cytometry of sciatic nerve tissue from mice reveal a significant infiltration of F4/80+ (p = 0.001) and CD11b+ cells (p = 0.002) into the sciatic nerve of Tg+ sick mice compared to Tg− mice. C, Flow cytometry of spinal cord tissue reveals an increase in F4/80+ cells (p = 0.002) into the spinal cord of Tg+ sick mice. Sciatic nerve sections (200X) from mice were stained with APC conjugated anti-F4/80 antibody reveal no infiltration of F4/80+ macrophages in D, wild-type or E, Tg+ healthy animals. Significant infiltration of macrophages is seen in F, Tg+ sick early mice. Decreased macrophages and loss of tissue are observed in sciatic nerves from G, Tg+ sick late mice.

To identify the cellular infiltrate observed in the sciatic nerve of Tg+ sick mice, tissues from Tg− and Tg+ sick mice were harvested and flow cytometry was used to identify various immune cells, including macrophages (F4/80), monocytes (Cd11b), T cells (CD3), and neutrophils (Gr1). Spleens from Tg+ sick mice revealed decreased macrophages (p<0.001) and increased T cell populations (p = 0.008) compared to Tg− littermates (Fig. 4A). The sciatic nerve of Tg+ sick mice revealed a significant infiltration of macrophages (p = 0.001) and monocytes (p = 0.002) compared to Tg− mice, but no difference in neutrophils (p = 0.469) was observed (Fig. 4B). These results suggest that there is recruitment of macrophages from the spleen to these tissues. Although not evident in H&E staining, spinal cord tissue from Tg+ sick mice had a significant infiltration of macrophages (p = 0.002) as well (Fig. 4C), which likely corresponds to cellular infiltration into the dorsal roots and not actual cord tissue.

Additionally, sciatic nerve tissue sections were stained with APC-conjugated anti-F4/80 antibody and examined by confocal microscopy. Tg− and Tg+ healthy mice exhibited no infiltration of macrophages into the sciatic nerve (Fig. 4D and E). Tg+ sick early mice exhibited abundant macrophages in the sciatic nerve, virtually infiltrating the entire tissue (Fig. 4F). Sciatic nerve tissue from Tg+ sick late mice demonstrated infiltrating macrophages (Fig. 4G), although to a much lesser degree than Tg+ sick early tissues. Again, tissue loss was evident in Tg+ sick late mice. This, combined with the above observation of macrophages infiltrating the sciatic nerve at the time of disease suggested that macrophage-mediated demyelination of the sciatic nerve and subsequent loss of tissue resulted in clinical disease.

**Figure 5:**
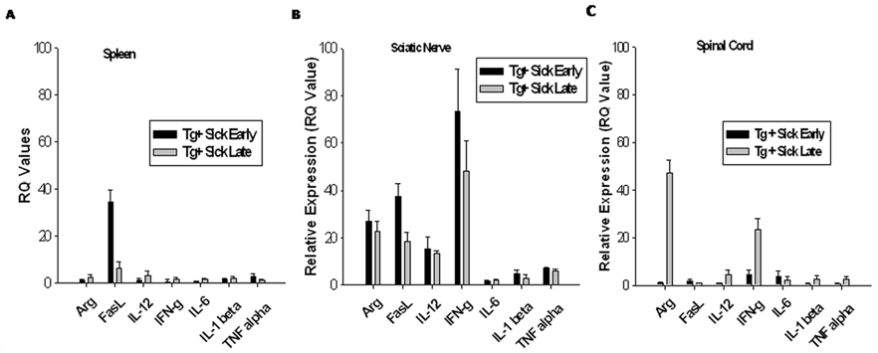
Inflammatory gene expression profiles of various tissues from VMD2-IL-10 transgenic mice. A, Spleens from Tg+ mice reveal relatively low expression of pro-inflammatory cytokines, with the exception of increased FasL in Tg+ sick early spleens. B, Sciatic nerve tissues, however, demonstrate multiple cytokines with increased gene expression, both pro-inflammatory (FasL, IL-12, IFN-g) and anti-inflammatory (Arg1). C, Spinal cord tissues have few cytokines with increased expression, and only at late stages of disease.

**Figure 6:**
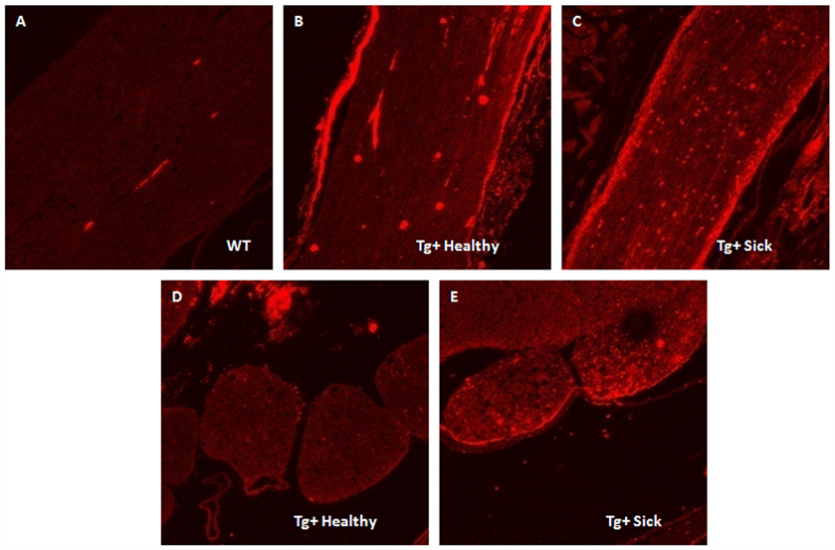
ICAM-1 expression in VMD2-IL-10 transgenic mice. Sciatic nerve sections from A, Tg− mice demonstrate no ICAM-1 expression, yet B, Tg+ healthy and C, Tg+ sick sciatic nerve sections reveal significant expression of ICAM-1 on the outer surface of the sciatic nerve. D, Spinal cord sections from Tg+ healthy mice reveal no expression of ICAM-1, yet the dorsal roots of E, Tg+ sick mice eveal positive staining of ICAM-1.

To determine the gene expression cytokine profile of various disease-affected tissues, spleen, sciatic nerve, and spinal cord tissue from Tg+ healthy and sick mice were harvested and examined for various pro- and anti-inflammatory cytokines by quantitative real-time PCR. Using Tg+ healthy tissues as a baseline comparison, Tg+ sick early and sick late spleens exhibited very little change in cytokine gene expression (Fig. 5A). Sciatic nerves, however, exhibited a primarily pro-inflammatory cytokine profile, with cytokine expression levels increasing with disease progression (Fig. 5B). These cytokines include FasL, IFN-γ, and IL-12. The anti-inflammatory marker arginase (Arg1) was also increased in sciatic nerve tissues from sick mice, suggesting a possible attempt to squelch this burst of autoimmune activity. Pro- and anti-inflammatory cytokine gene expression is also evident in spinal cord tissue of Tg+ sick mice (Fig. 5C), albeit at drastically reduced levels compared to the sciatic nerve tissue. Pancreas specific IL-10 transgenic mice have been shown to develop spontaneous inflammation and diabetes [9], [10]. Cellular infiltration into the pancreas correlated with increased expression of adhesion molecules on pancreatic endothelium, including intercellular adhesion molecule 1 (ICAM-1) [10]. To determine if VMD2-IL-10 Tg mice exhibited increased ICAM-1 expression, sciatic nerve and spinal cord tissues sections were stained with PE-conjugated anti-ICAM-1 antibody. Tg− littermates exhibit no expression of ICAM-1 on sciatic nerve tissue sections (Fig. 6A), whereas Tg+ healthy and sick mice demonstrated significant staining of ICAM-1 antibody on the surface of the sciatic nerve tissue (Fig. 6B and C). Increased ICAM-1 expression was not evident on spinal cord dorsal roots in Tg+ healthy mice (Fig. 6D), but was evident once Tg+ mice develop disease (Fig. 6E). No increase in ICAM-1 expression was observed in the cerebellum of Tg−, Tg+ healthy, or Tg+ sick mice (data not shown), reflecting that sites spared of cellular infiltration lack ICAM-1 expression.

**Figure 7:**
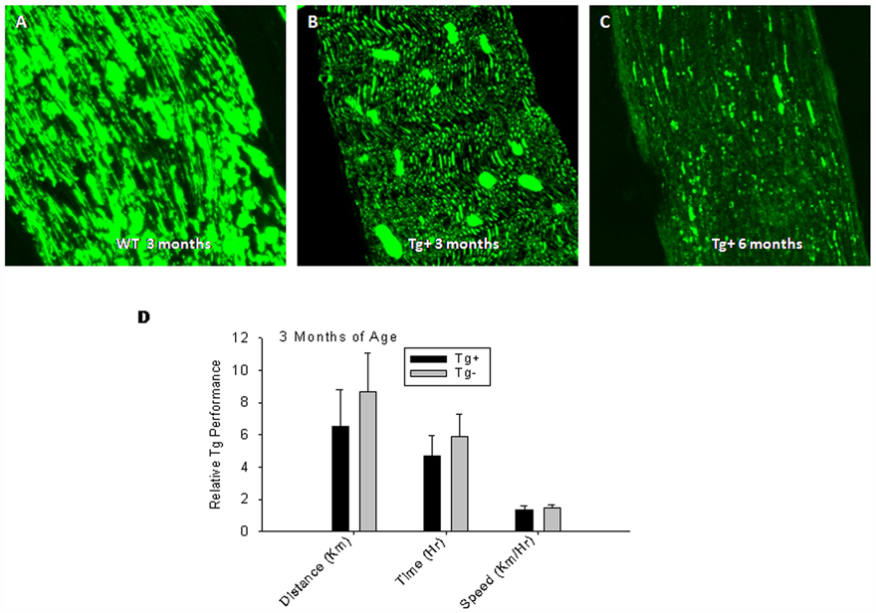
Neural phenotype of VMD2-IL-10 transgenic mice. A, Tg− sciatic nerve sections are strongly reactive with the SMI-31 antibody, indicating normal neurofilament phosphorylation and phenotype. B, Sciatic nerve sections from 3 month-old Tg+ mice demonstrate decreased reactivity with SMI-31, indicating a decreased loss of neurofilament in the early stages of disease prior to observable levels of cellular infiltration. C, Tg+ sick sciatic nerve sections, however, demonstrate even less reactivity with SMI-31, indicating dephosphorylation of neurofilaments and neural loss correlating with cellular infiltration. D, Voluntary wheel running (VWR) experiments with Tg+ mice (n = 3) before cellular infiltration and disease onset (3-months old) reveals no loss of motor function (distance p = 0.320; time p = 0.341; speed p = 0.576) prior to macrophage infiltration compared to age-matched Tg− littermates (n = 3).

In our VMD2-IL-10 transgenic mice, it appears that disease progression correlated with infiltration of macrophages into the sciatic nerve and subsequent demyelination. However, paralysis can be initiated by a variety of mechanisms other than antecedent demyelination. In addition, although mice did not clinically demonstrate evidence of weakness or paralysis prior to demyelination, it remained possible there was some subclinical axonal dysfunction prior to initiation of demyelination. This includes inherent neuronal defects resulting in neuromuscular dysfunction. In order to determine if Tg+ mice have underlying axonal dysfunction prior to development of paralysis, we first stained sciatic nerve tissue sections with an antibody specific for normal neurofilament phenotype. The SMI-31 antibody reacts with a phosphorylated epitope in extensively phosphorylated neurofilament H and, to a lesser extent, with neurofilament M, a sign of normal neurofilament status. SMI 31 reacts broadly with thick and thin axons and some dendrites such as basket cell dendrites, but not Purkinje cell dendrites. Nerve cell bodies are generally unreactive, and other cells and tissues are unreactive except for peripheral axons. Damage to neurofilaments results in dephosphorylation of neurofilaments, and thus less reactivity with SMI-31. Wild-type mice demonstrated significant positive staining with SMI-31, reflecting a healthy axonal phenotype (Fig. 7A). Tg+ mice developed progressively decreased staining with SMI-31 as they developed weakness and paralysis reflecting axonal damage and loss with onset of paralysis (Fig. 7B and C).

We next examined whether the loss of neurofilament staining observed in Tg+ mice correlated with a loss of activity at times preceding macrophage infiltration and hind limb paralysis. This was done by examining voluntary wheel running (VWR) of Tg+ mice and their Tg− age-matched littermates. Three month-old Tg+ mice, prior to developing clinical signs of weakness or motor dysfunction, showed no significant difference in VWR compared to Tg− littermates, as determined by distance, time, and speed on the wheel (Fig. 7D). This demonstrates that although mice at 3 months of age demonstrate decreased neurofilament staining (Fig. 7B), loss of function in these animals does not progress until cellular infiltration and demyelination occurs, indicating that macrophages are the primary mediators of disease in Tg+ mice. This also demonstrates that paralysis and/or loss of activity is not due to an inherent neurological dysfunction in Tg+ mice.

Disease progression in VMD2-IL-10 transgenic mice appears to develop in two stages. First, overexpression of IL-10 results in upregulation of ICAM-1 on sciatic nerve tissue. Secondly, macrophages infiltrate sciatic nerve tissue, cause demyelination, neuronal dysfunction, and ultimately paralysis. We wanted to test whether the onset of disease could be delayed or disease severity ameliorated by attempting to remove important components of disease progression: IL-10 and macrophages. We first attempted to block IL-10 protein expression with blocking antibodies to IL-10. Tg+ mice were treated with intraperitoneal injections of 250 µg anti-IL-10 antibody or rat IgG control antibody i.p. 1–2x weekly. Anti-IL-10 immunotherapy did not successfully delay progression of disease compared to isotype control administration (Fig. S2I). Higher doses of anti-IL-10 were not successful and resulted in toxicity (data not shown). The lack of success with i.p. administration of anti-IL-10 was not surprising, as systemic levels of IL-10 were not elevated in transgenic mice (Fig. 2B). Moreover, systemically delivered antibodies would not readily cross blood-brain barriers and penetrate nerve tissues.

**Figure 8:**
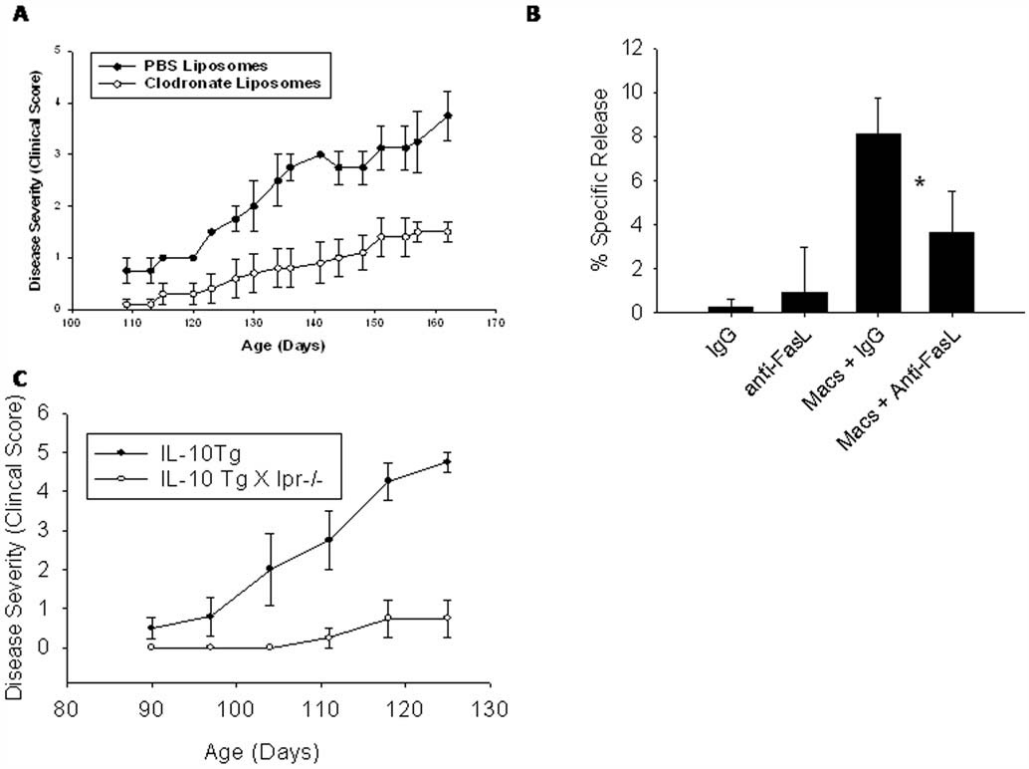
Macrophage mediated disease and Fas ligand-mediated cytotoxicity of myelin-producing Schwann cells in VMD2-IL-10 transgenic mice. A, Depletion of macrophages in Tg+ mice by administration of clodronate liposomes i.p. twice weekly (n = 6) results in significant delayed onset of disease compared to PBS liposome-treated Tg+ mice (n = 5). B, Tg+ macrophages and Schwann cells were co-cultured in the presence of anti-FasL antibodies or isotype control. Cytotoxicity of Schwann cells is observed with macrophages + IgG, yet cytotoxicity is significantly decreased when FasL is blocked *(p = 0.0035). Antibodies alone do not induce Schwann cell cytotoxicity. C. VMD2-IL-10 Tg+ mice were bred with C57BL/6-Fas^lpr^ mice, which harbor a spontaneous mutation in Fas that prevents Fas-FasL interaction and signaling. Tg+ *lpr* homozygous mice failed to develop progressive disease and paralysis compared to Tg+ mice.

We next attempted to ameliorate disease by depletion of macrophages with clodronate liposomes. Tg+ mice were treated with intraperitoneal injections of clodronate or PBS liposomes (200 µl initial injection; 100 µl each subsequent injection) twice weekly [11]. Liposome treatment commenced immediately prior to onset of disease (day 100). Tg+ mice treated with clodronate liposomes developed paralysis at a significantly reduced tempo compared to PBS liposome-treated Tg+ mice (Fig. 8A). Clodronate liposome treatment was continued for 2 months, and ceased at day 162 due to toxicity related to repeated liposome injection. At time of cessation, the clinical score of paralysis was 1.5±0.41 for clodronate treated mice compared to 3.75±0.96 for PBS-treated mice. PBS liposome-treated Tg+ mice continued to live past day 162, suggesting that toxicity is directly attributable to the clodronate and not to liposomes. Clodronate-liposome treatment resulted in significantly reduced macrophage infiltrate into the sciatic nerve (Fig. S2H).

It was previously observed that gene expression of FasL was upregulated in the spleen and sciatic nerve of Tg+ mice (Fig. 4A and B). Since membrane bound FasL can regulate cell death [1], [3], [12], we hypothesized that this increase in FasL may be due to increased expression on Tg+ macrophages, and that these macrophages are utilizing FasL to mediate myelin-producing Schwann cell death in the sciatic nerve, leading to demyelination of the sciatic nerve and ultimately paralysis of the animal. To determine if Tg+ macrophages utilize FasL to induce Schwann cell death, a modified macrophage/target-cell co-culture experiment was utilized [13]. Macrophages from Tg+ sick mice were isolated and co-cultured with radioactively labeled murine Schwann cells at an effector∶target ratio of 10∶1. Schwann cell death was determined by the release of ^3^H-thymidine into the cell supernatant. The co-culture was performed with anti-FasL blocking antibodies or isotype control antibodies present. Tg+ macrophages induced significant Schwann cell cytotoxicity, and was due to FasL mediated death, as blocking antibodies to FasL significantly inhibited Schwann cell death (Fig. 8B). Therefore, macrophages-mediated cytotoxicity of myelin-producing Schwann cells through FasL-dependent pathways. To determine if FasL was indeed causing demyelination and paralysis *in vivo*, we bred VMD2-IL-10 Tg+ mice with C57BL/6-Fas^lpr^ mice, which harbor a spontaneous mutation in Fas that prevents Fas-FasL interaction and signaling. We found that Tg+ *lpr* homozygou*s* mice failed to develop progressive disease and paralysis compared to Tg+ mice (Fig. 8C), confirming that Fas expression on neural tissues is necessary for macrophage- and FasL-mediated demyelination and paralysis in Tg+ mice.

## Discussion

In conclusion, VMD2-driven transgenic expression of IL-10 results in macrophage recruitment and FasL-mediated demyelination of the sciatic nerve, axonal death, and ultimately paralysis. Although IL-10 is an immunosuppressive cytokine at normal levels, overexpression of IL-10 exacerbates inflammation. IL-10 upregulates the adhesion molecules ICAM-1, that causes increased trafficking of inflammatory macrophages into the sciatic nerve via CD11b expressed on the macrophage surface, a receptor for the ligand ICAM-1[14].

Macrophages and sciatic nerve tissue in Tg+ sick mice exhibit a markedly pro-inflammatory gene expression profile, as evident by upregulated expression of FasL, IFN-γ, and IL-12. This contrasts with the known effect of IL-10 as an immunosuppressive cytokine that polarizes macrophages towards an anti-inflammatory and pro-angiogenic gene expression profile [1], [2]. Cytokines observed at these timepoints provide an overall “snapshot” of the cytokine milieu of Tg+ sick mice at early and late stages. Also, comparisons with Tg+ healthy mice rather than Tg− mice provide a representation of disease progression in Tg+ mice rather than comparing Tg+ mice with a wild-type phenotype. Increased expression of FasL may be the cause of demyelination and tissue loss, as Tg+ macrophages induced death of myelin-producing Schwann cells in a FasL-dependent manner, and Tg+ *lpr* homozygous mice failed to develop progressive disease.

It has been considered that IL-10 may be a potential therapeutic in the treatment of autoimmune diseases [15]. Administration of recombinant IL-10 (rIL-10) reduces disease severity in experimental models of diabetes, rheumatoid arthritis, and inflammatory bowel disease [16], [17], [18]. However, the outcome of rIL-10 therapy in autoimmune diseases of the nervous system is unclear. In models of experimental autoimmune encephalomyelitis (EAE), intravenous injection of rIL-10 exacerbated disease [19], while subcutaneous administration of rIL-10 reduced disease severity [20]. Targeting delivery of IL-10 to the central nervous system does not increase the success of IL-10 treatment, as intracranial injection of soluble rIL-10 or plasmids encoding IL-10 did not suppress EAE [21]. Therefore, administration and/or localization of IL-10 may be critical in determining its immunoregulatory function in autoimmune diseases of the nervous system.

In our VMD2-IL-10 transgenic animals, IL-10 is not only upregulated in the sciatic nerve, but also the eye, brain, spinal cord, and especially the testes. However, our disease phenotype is restricted primarily to the sciatic nerve, and to a much lesser extent the caudal spinal cord. One hypothesis for why other organs are spared immune mediated destruction may be the immune privileged environment that these tissues possess. The eye, brain, and spinal cord are well known immune privileged sites, and possess various anti-inflammatory and pro-apoptotic molecules that may dampen an immune response in these tissues [22], [23]. Also, as the disease progresses from early stages to late stages, cellular infiltration and/or demyelination may be ascending from lower limbs to upper extremities, similar to human CIDP. This may explain while caudal spinal cord and brachial plexus nerves are not affected until later stages of disease. However, the effect of cellular infiltration into these tissues at these later stages on disease phenotype is not fully understood.

Polyneuropathies are a common neurological disease that affects the peripheral nervous system in humans. The use of animal models has helped to recapitulate and understand human neuropathies, however the transfer of treatment modalities from preclinical to clinical settings has been highly unsuccessful [24]. This may be due to the fact that established animal models do not precisely mimic human disease. For example, experimental autoimmune neuritis (EAN), the most widely used model of Guillain-Barré syndrome (GBS), involves the immunization of rats with peripheral myelin homogenates, myelin proteins, or derived peptides to produce disease [24]. This results in a primarily T cell mediated attack and demyelination of the peripheral nerves. GBS, however, results from macrophages serving as the primary effector cells in the demyelination of the peripheral nervous system. The VMD2-IL-10 transgenic mouse model may be a useful mouse model for the study of GBS pathogenesis and treatment. Animal models of CIDP have been extremely problematic in mimicking human disease. EAN models are acute, with disease recovery occurring substantially by 28 days. Other animal models for CIDP involve repeated myelin immunization that may be combined with immunosuppression [24]. These models are not ideal, as they are primarily performed in species other than mice, hampering the use of genetic knockout animals.

Interestingly, a mouse model of CIDP has been previously demonstrated in B7-2 deficient non-obese diabetic (NOD) mice [25]. This animal model results in the infiltration of T cells and dendritic cells into the peripheral nervous system. However, disease progression was not congruent between both sexes, and cellular infiltration lacked macrophages as observed in human CIDP. In addition, autoimmune disease in these mice was manifested on two genetic backgrounds with preexisting immunodeficiencies. VMD2-IL-10 transgenic mice may be an ideal mouse model for the study of the pathobiology of CIDP and may be an excellent surrogate to investigate therapeutic options in this disease.

## Materials and Methods

### Ethics Statement

All work was carried out in accordance with Association for Research in Vision and Ophthalmology (ARVO) guidelines. Comprehensive protocols of animal care and experimental design outlined in this study are on file and have been approved by the Washington University Committee on the Humane Care of Laboratory Animals (CHCLA). All animal caretakers and laboratory personnel have appropriate approvals based on specific AALAC-approved training programs. The facilities are inspected at regular intervals by a local University Committee and by unannounced visits directed by the Federal Government.

### Mice

VMD2-IL-10 transgenic (Tg) mice were constructed to overexpress IL-10 (*pVMD2-placF* was provided by Noriko Ezumi, Johns Hopkins Medical School). The human *VMD2* promoter was removed and cloned into the pCI plasmid (Promega), replacing the CMV promoter, and then placed the IL-10 ORF downstream [3]. Tg mice were produced by injecting fertilized mouse oocytes with transgene DNA by standard protocols in the Washington University Department of Ophthalmology and Visual Science Molecular Biology Core facility. B6CBAF2 founders were screened by PCR and used for backcrossing to C57BL/6 mice. IL-10 expression was verified by RT-PCR (forward – 5′ GGA CTT TAA GGG TTA CTT GGG TTG CC 3′; reverse – 5′ CAT TTT GAT CAT CAT GTA TGC TTC T 3′). Transgene-negative littermates were used as wild-type control mice.

C57BL/6-Fas*^lpr^* mice were purchased from Jackson Laboratories (Bar Harbor, ME) and bred with VMD2-IL-10 Tg+ mice. The resulting F1 offspring were screened for *lpr* (according to Jackson Laboratory instructions) and Tg+ (as described above) status, and Tg+ *lpr* heterozygous mice were bred. F2 generation Tg+ *lpr* homozygous offspring were identified by PCR and monitored for disease progression.

### Clinical score of paralysis

Disease severity was scored as: grade 0, normal; grade 1, partial paralysis of 1 hind limb; grade 2, complete paralysis of 1 hind limb; grade 3, partial paralysis of both hind limbs; grade 4, complete paralysis of both hind limbs; grade 5, moribund or death. Data is reported as mean±SEM.

### Immunohistochemistry

5-µm paraffin embedded sections were stained with Hematoxylin and Eosin (H&E), Luxol fast blue myelin specific stain, allophycocyanin (APC)-conjugated anti-mouse F4/80 (BM8; eBioscience), phycoerythrin (PE)-conjugated anti-mouse CD54 (anti-ICAM-1; N1/1.7.4; eBioscience), PE-conjugated Rat anti-mouse IL-10 (BD Biosciences), or mouse anti-phosphorylated neurofilament antibody (SMI-31; Covance) followed by fluorescein isothiocyanate (FITC)-conjugated goat anti-mouse IgG1 (Caltag). All antibodies were used at 1 µg/ml. Staining was examined by confocal microscopy (Zeiss LSM 510).

### Flow cytometry

Single-cell suspensions from spleens, sciatic nerves, and spinal cords from wild-type and transgene-positive sick mice were obtained and stained with PE-conjugated IgG2a (eBM2a; eBioscience), PE-conjugated anti-mouse F4/80 (BM8; eBioscience), FITC-conjugated anti-mouse CD3e (1 45-2C11; eBioscience), or FITC-conjugated anti-mouse Cd11b (M1/70; BD Biosciences). Cells were then assessed for fluorescence in a FACScan flow cytometer (BD Biosciences) and the results were analyzed using Cellquest software (BD Biosciences).

### Enzyme-linked immnosorbent assay (ELISA)

Cerebrospinal fluid (CSF) was collected from the cisterna magna[26], and plasma was sampled via cardiac bleed from wild-type, transgenic healthy, and transgenic sick mice. CSF and plasma samples were then assayed for IL-10 protein levels via mouse IL-10 Quantikine ELISA kit (R&D Systems). CSF and plasma from 3 separate animals for each group were assayed.

### Quantitative Analysis of Gene Expression (Quantitative Real-Time RT-PCR)

Relative expression of various inflammatory proteins were performed as previously described[1]. Tissues examined were whole spleens, sciatic nerve tissue, and spinal cord tissue from VMD2-IL-10 Tg + healthy, early sick (grade <3), and late sick (grade ≥3) mice.

Taqman Gene Expression Assay Mixes used (primer/probe sets) include β-actin (Mm00607939_s1); TNF-α (Mm00443258_m1); IL-6 (Mm00446190_m1); IL-12 (Mm01288989_m1); FasL (Mm00438864_m1); IL-1β (Mm00434228_m1); IFN-γ (Mm00801778_m1); and Arg1 (Mm00475988_m1). Tissues from transgene-positive healthy mice were used as a baseline comparison

### Voluntary Wheel-Running (VWR)

Mice were tested after being segregated to individual cages with a voluntary exercise wheel recorded by an automatic speedometer. After a 5-day acclimation period, two 24 h running periods were recorded for speed, total distance, and total time on the wheel.

### *In vivo* anti-IL-10 antibody treatment

VMD2-IL-10 Tg + mice were treated with intraperitoneal injections of rat anti-mouse IL-10 blocking antibody (250 µg; JES5.2A5; Genzyme) or Rat IgG (250 µg; Equitech-Bio). Injections were administered 1–2 times weekly beginning on day 50. Paralysis was monitored upon day of injection.

### Liposome-encapsulated dichloromethylene diphosphonate

Multilamellar liposomes were prepared as described previously [27]. The cytotoxicity of C12MDP-LIP and PBS-LIP were tested for in vitro toxicity against RAW 264.7 macrophages before use. Liposomes were stored at 4°C for up to 1 month.

### *In vitro* macrophage-mediated cytotoxicity assay

Utilizing a modified version of a macrophage/target cell cytotoxicity assay from previously published reports [13], the murine Schwann cell line, SW10 (ATCC), was grown at the proliferative temperature of 33°C. SW10 cells were trypsinized, washed, and resuspended in DMEM media with 8 μCi ^3^H-thymidine add per 10 ml media. Cells were then plated in 96-well round-bottom plates at 1×10^4^ cells/well at 37°C and allowed to adhere for 24 hours. This temperature prevents Schwann cell proliferation but induces myelin production [28]. Splenic macrophages from Tg+ sick mice were isolated by magnetic separation as described above. Schwann cells were washed and macrophages were added and allowed to co-culture for 24 hours. After incubation, cells were lightly centrifuged and 100 µl of supernatant was removed and placed in 2 ml scintillation fluid. Spontaneous release was determined by Schwann cells incubated with no macrophages, and maximum release was determined by lysis of cells with Qiagen lysis buffer RLT. Specific release was determined by the equation:
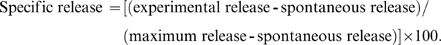



Co-cultures of macrophages and Schwann cells were performed in the presence of blocking antibodies to FasL or Hamster IgG (10 µg/ml; eBioscience). Also, Schwann cells alone were incubated with antibodies to ensure no antibody-mediated cytotoxicity.

Cytotoxicity data from quintuplicate cultures are presented as the mean±SD.

### Statistics

A student's *t* test was performed for statistical analysis. A p value of <0.05 was considered significant.

## Supporting Information

Figure S1 IL-10 gene and protein expression in VMD2-IL-10 transgenic mice. A, Tg- and B–C, Tg+ mice reveal increased IL-10 expression on the outer surface of the sciatic nerve. Spinal cord sections (200X) from D, Tg- and E-F, Tg+ reveal increased IL-10 on the dorsal roots of Tg+ mice only after development of disease.
(0.45 MB TIF)

Figure S2 Additional peripheral nerve sections and anti-IL-10 treatment. No immune cell infiltration is observed in the A, brachial plexus of Tg − or B, Tg+ healthy mice. Cellular infiltration is observed in Tg+ sick mice C, despite the lack of disease phenotype in the forelimbs. Immune cell infiltrate is not observed in the femoral nerves of D, Tg−, E, Tg+ healthy, and F, Tg+ sick mice. G, Optic nerve sections from Tg+ sick late mice do not reveal infiltration of immune cells despite increased VMD2 and IL-10 expression. H, Sciatic nerve sections from clodronate-liposome treated Tg+ mice at day 162 reveal cellular infiltrate, yet at significantly decreased levels. I, Anti-IL-10 immunotherapy (250 mg i.p. twice weekly) of Tg+ mice (n = 4) does not decrease disease onset compared to isotype control treated Tg+ mice (n = 3).
(0.73 MB TIF)

Table S1 Summary of cellular infiltration and disease phenotype in various nervous tissues from VMD2-IL-10 Tg− and Tg+ mice.
(0.04 MB DOC)
