# Supplementary figures and images for: Interleukin-10 Overexpression Promotes Fas-Ligand-Dependent Chronic Macrophage-Mediated Demyelinating Polyneuropathy
Source: PLoS One. 2009 Sep 22;4(9):e7121. doi: 10.1371/journal.pone.0007121 (PMC2743195; doi:10.1371/journal.pone.0007121)

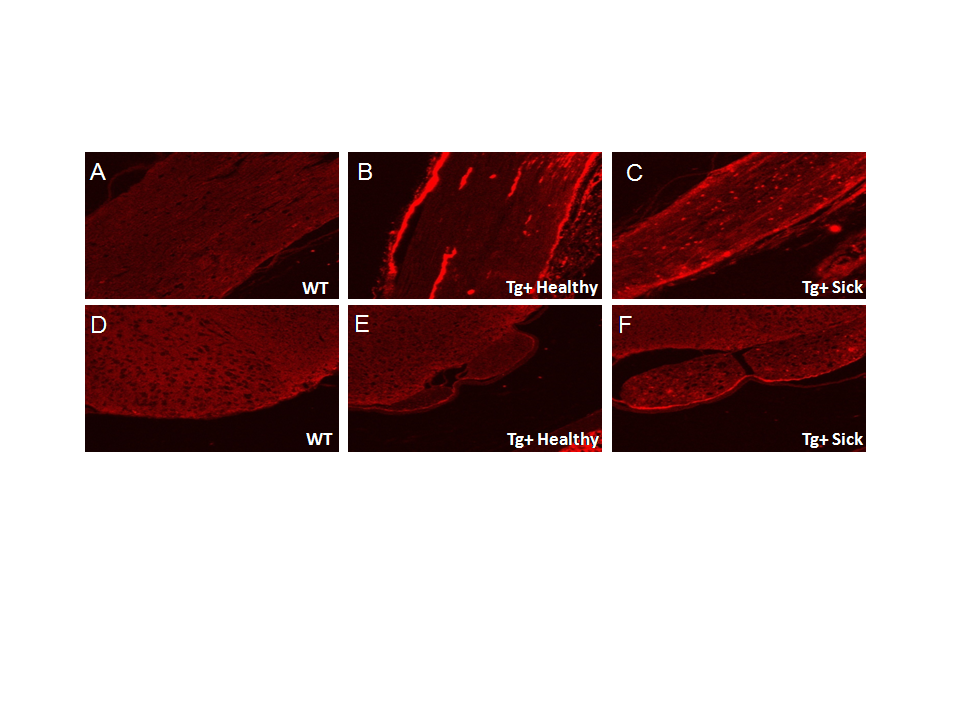

Supplement: Figure S1 — IL-10 gene and protein expression in VMD2-IL-10 transgenic mice. A, Tg- and B–C, Tg+ mice reveal increased IL-10 expression on the outer surface of the sciatic nerve. Spinal cord sections (200X) from D, Tg- and E-F, Tg+ reveal increased IL-10 on the dorsal roots of Tg+ mice only after development of disease. (0.45 MB TIF) [file pone.0007121.s001.tif]

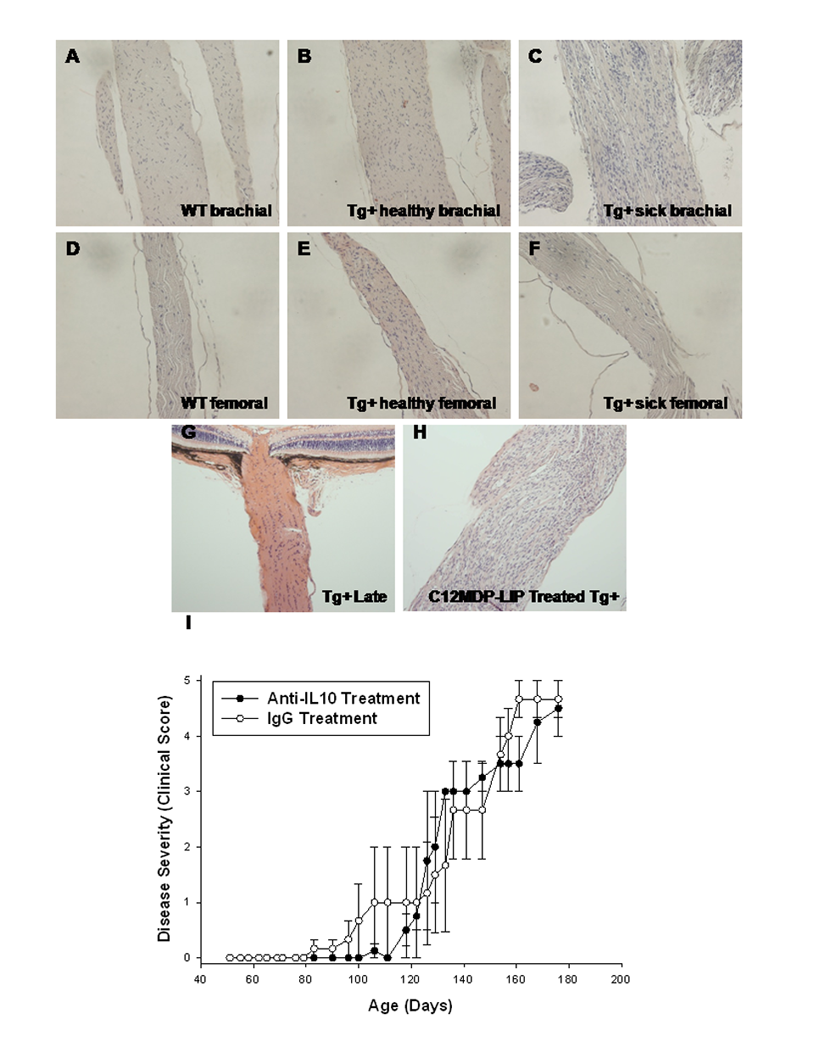

Supplement: Figure S2 — Additional peripheral nerve sections and anti-IL-10 treatment. No immune cell infiltration is observed in the A, brachial plexus of Tg − or B, Tg+ healthy mice. Cellular infiltration is observed in Tg+ sick mice C, despite the lack of disease phenotype in the forelimbs. Immune cell infiltrate is not observed in the femoral nerves of D, Tg−, E, Tg+ healthy, and F, Tg+ sick mice. G, Optic nerve sections from Tg+ sick late mice do not reveal infiltration of immune cells despite increased VMD2 and IL-10 expression. H, Sciatic nerve sections from clodronate-liposome treated Tg+ mice at day 162 reveal cellular infiltrate, yet at significantly decreased levels. I, Anti-IL-10 immunotherapy (250 mg i.p. twice weekly) of Tg+ mice (n = 4) does not decrease disease onset compared to isotype control treated Tg+ mice (n = 3). (0.73 MB TIF) [file pone.0007121.s002.tif]
