# Supplementary material for: Interleukin-10 Overexpression Promotes Fas-Ligand-Dependent Chronic Macrophage-Mediated Demyelinating Polyneuropathy
Source: PLoS One. 2009 Sep 22;4(9):e7121. doi: 10.1371/journal.pone.0007121 (PMC2743195; doi:10.1371/journal.pone.0007121)
Supplement: Table S1 — Summary of cellular infiltration and disease phenotype in various nervous tissues from VMD2-IL-10 Tg− and Tg+ mice. (0.04 MB DOC) [file pone.0007121.s003.doc]

| **Tissue** | **Diseae Stage** | **Cellular Infiltration Observed** | **Demyelination or Paralysis Observed** |
| --- | --- | --- | --- |
| Sciatic Nerve | WT | N | N |
|  | Tg+ Healthy | N | N |
|  | Tg+ Sick Early | Y | Y |
|  | Tg+ Sick Late | Y | Y |
| Spinal Cord | WT | N | N |
|  | Tg+ Healthy | N | N |
|  | Tg+ Sick Early | N | Y (Dorsal Roots) |
|  | Tg+ Sick Late | N | Y (Dorsal Roots) |
| Brachial Plexus | WT | N | N |
|  | Tg+ Healthy | N | N |
|  | Tg+ Sick | Y | N |
| Femoral Nerve | WT | N | N |
|  | Tg+ Healthy | N | N |
|  | Tg+ Sick | N | N |
| Optic Nerve | Tg+ Sick Late | N | N |
